# Echocardiographic imaging and ventricular mechanics in pulsatile-flow LVAD pediatric patients: a systematic approach

**DOI:** 10.3389/fped.2024.1345891

**Published:** 2024-01-29

**Authors:** R. Iacobelli, A. Di Molfetta, A. Amodeo

**Affiliations:** ^1^ Pediatric Cardiology Unit, Clinical Area of Fetal and Cardiovascular Science, Bambino Gesù Children’s Hospital, Rome, Italy; ^2^ Cathetherization Laboratory, Fabrizio Spaziani Hospital, Frosinone, Italy; ^3^ Heart Failure Unit, Cardiac Transplantation and MCS, Clinical Area of Fetal and Cardiovascular Science, Bambino Gesù Children’s Hospital, Rome, Italy

**Keywords:** echocardiography, children, pulsatile-flow LVAD, ventricular mechanics, heart-LVAD interaction

## Abstract

Echocardiography plays a crucial role in determining the eligibility for left ventricular assist device (LVAD) placement in patients experiencing advanced heart failure (HF) and in monitoring patient care after the implantation procedure. Because of its unique nature, pediatric population and pulsatile-flow LVADs used in pediatrics require specific skills so that pediatric echocardiographers must develop a systematic approach in order to image the patients pre and post LVAD implantation. Therefore, the purpose of this narrative review is to delineate a systematic echocardiographic approach for pediatric patients supported by pulsatile-flow LVADs.

## 1 Introduction

The use of LVADs is a widely accepted therapeutic approach for supporting pediatric patients as a bridge to cardiac transplantation, for decision-making, as destination therapy, or as a bridge to recovery (1). Echocardiography represents a crucial tool for identifying patients who require LVAD and is an optimal modality for post-implantation monitoring due to its non-invasive nature and widespread availability at bedside. Both Transthoracic and Transesophageal Echocardiography (TTE, TOE) play a fundamental diagnostic role in imaging patients receiving LVADs (2–8).

Although reports from adult patients with LVADs have shown the invaluable role of the echocardiographic evaluation in a wide range of devices (2, 8, 9), this information may not be entirely applicable to the pediatric population due to the broader heterogeneity of anatomical diagnoses in children, the wider size range among pediatric patients, and the use of different device types specifically designed for children.

Despite the increasing use of long-term continuous-flow devices in bigger and older children, implantation of a para-corporeal pulsatile-flow device remains the unique option for long-term support in small pediatric patients with BSAs that prohibit the use of intra-corporeal device (10).

To our knowledge, greater interest is placed on advancing innovative techniques to assess the interaction between the native heart and continuous flow VADs, primarily employed in adults (16). However, there is a shortage of data concerning the evaluation of pulsatile flow VADs, such as the Berlin Heart EXCOR VAD (BH), mainly used in the pediatric population (11). Consequently, our objective is to underscore the evolving clinical use of echocardiography in assessing ventricular structure and function, hemodynamics, valvular function, native heart-LVAD interaction, right and left ventricular mechanics and deformation and ultimately myocardial recovery in the pediatric population developing an echocardiographic systematic approach and clinical protocol to Berlin Heart EXCOR LVAD pediatric patients (Table 1).

**Table 1:** Aim of echocardiographic evaluation during LVAD support in children.

Assessment of ventricular function with echocardiographic conventional indices
Assessment of hemodynamics during support
Study of valve function
Assessment of interaction between native heart and LVAD
Assessment of RV and LV mechanics and deformation (STE)
Assessment of myocardial recovery

RV: right ventricular; LV: left ventricular; STE: speckle-tracking echocardiography.

## 2 Berlin heart EXCOR functioning

The Berlin Heart (BH) is a para-corporeal, pneumatic, and pulsatile-flow ventricular assist device (VAD) system, comprising an inflow cannula, an outflow cannula, and a pneumatic chamber. Designed for left ventricular support (LVAD), right ventricular support (RVAD) and biventricular support (BiVAD), its cannulas and blood pumps are available in various sizes to support pediatric patients as small as 3 kg to adult-size teenagers.

**Figure 1:**
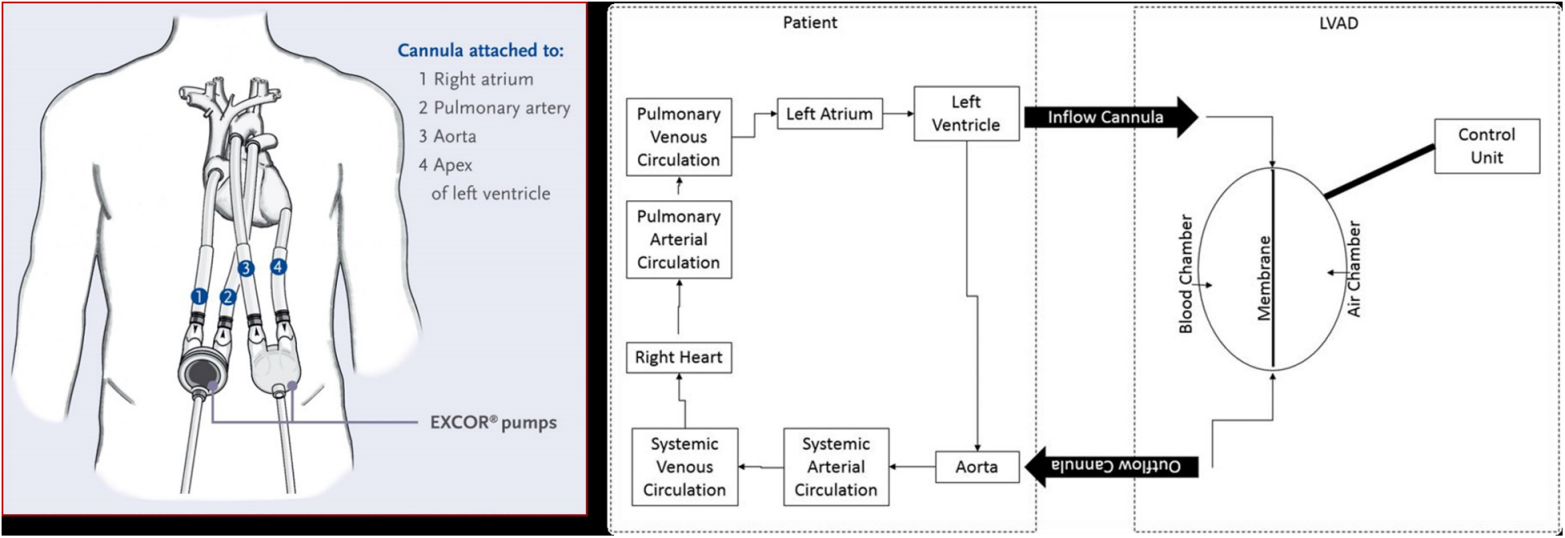
Left panel: Berlin heart paracorporeal excor pump configuration for biventricular support. Right panel: scheme illustrating BH LVAD components and connection to aorta and LV.

Silicone cannulas establish the connection between the blood pump and the patient, and tri-leaflet inflow and outflow valves, resembling semilunar valves, prevent blood regurgitation (Figure 1). All surfaces in contact with blood are coated with heparin. The polyurethane pneumatic chamber is divided into two parts by a three-layer flexible membrane, separating the blood from the air chamber. The membrane's movement is ensured by the connection between the pneumatic chamber and the driving unit (IKUS). It is possible for the operator to set parameters such as the VAD rate (30–150 bpm), the percentage of VAD systole duration (20%–70%), the VAD filling pressure (from −100 mmHg to 0), and ejection pressure (60–350 mmHg). While these parameters are typically suggested by the Berlin Heart EXCOR VAD manual, adjustments can be made to ensure the complete emptying or filling of the Berlin Heart EXCOR VAD chamber.

Typically, the patient's native heart and the Berlin Heart EXCOR VAD operate asynchronously, resulting in a BH rate different from the patient's heart rate. This disparity gives rise to the beat phenomenon, marked by the asynchronous opening of the aortic valve and the ejection of the BH.

The beat phenomenon is a derived concept from physics that occurs when two waves with different frequencies overlap, causing a cycle of alternating interference between waves. This happens because of the intrinsic nature of pulsatile-flow devices which operates in an asynchronous way with the cardiac cycle of the native heart. This turbulence is transmitted to both the Berlin Heart and the patient's cardiocirculatory system, as can be assessed by echocardiography (9).

As previously indicated (2, 4), the echocardiographic evaluation of individuals undergoing LVAD implantation should encompass morphological and physiological parameters to identify the benefits but also the potential risks of the procedure. Selecting optimal candidates is one of the major factor influencing both immediate surgical outcomes and long-term follow-up success.

In children, we can recognize five main phases of echocardiographic monitoring: (1) the patient selection for LVAD implantation performed by TTE, (2) the peri-operative phase during and post-implantation (guided by TOE); (3) LVAD standard surveillance and/or optimization exam (performed at pre-specified time interval); (4) problem-focused or troubleshooting echocardiographic examination (TTE or TOE) and (5) echocardiographic study during weaning protocols. See Table 2 for the complete check-list evaluation during LVAD support.

**Table 2:** Summary of pre, peri and post-implantation check list in pediatric patients supported by LVAD.

Pre-implanation check-list	Peri-implanation phase check-list	Post-implanation check-list
Cardiac morphology (any CHD)	Inflow&outflow cannula position	LV function (EF, EDD, LV-GLS)
LV function (EF, EDD, LV-GLS)	RV function	RV function
RV function (TAPSE, RVFAC, S', RAP, RVFW-LS, RVEDD/LVEDD ratio)	IVS position	IVS position
Value function (TR, MR, AR)	Value function (TR, MR, AR & AoV opening)	Value function (TR, MR, AR) & AoV opening
Intracardiac shunts and/or thrombosis	Intracardiac shunts & complete dearing	Inflow&outflow cannula position, thrombosis/obstruction

### 2.1 Pre implantation evaluation

Echocardiography to assess the structure and function of the heart before LVAD implantation is essential in children and serves two primary objectives: (1) evaluating the appropriateness of LVAD treatment for patients with HF, and (2) identifying cardiac abnormalities that may indicate potential complications following surgery (2–4).

All of these evaluations should be overseen and interpreted by a proficient echocardiographer with expertise in advanced HF and the hemodynamic evaluation of mechanical cardiac support. Special attention should be given to identifying findings commonly referred to as “red flags” and considering that after implantation we will have new echocardiographic views which are the inflow and the outflow graft of the device (4).

**Figure 2:**
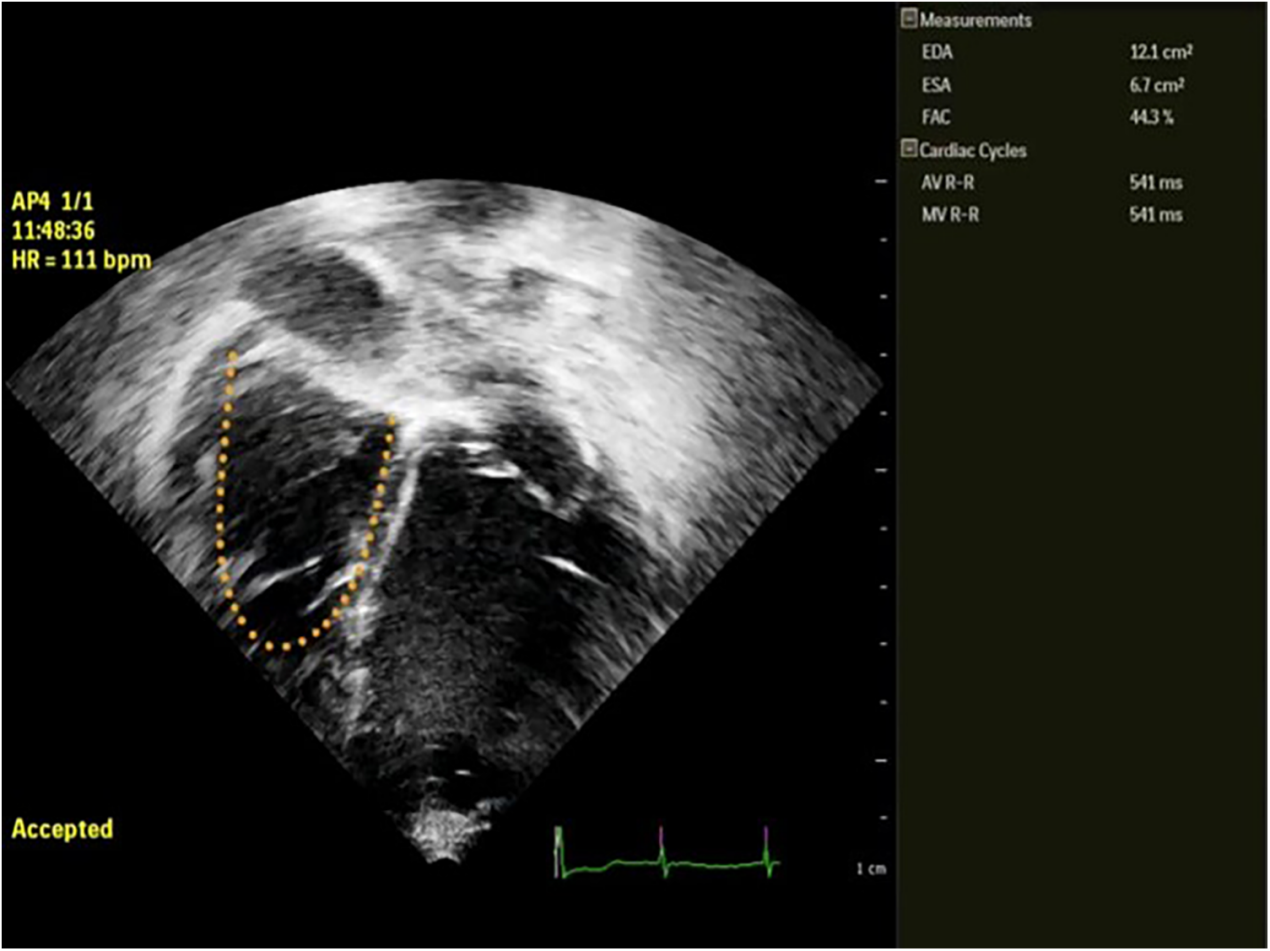
Semi automatic measurement of RVFAC in a pediatric patient with LVAD BH. EDA, end diastolic area; ESA, end systolic area; FAC, fractional area change in %.

**Figure 3:**
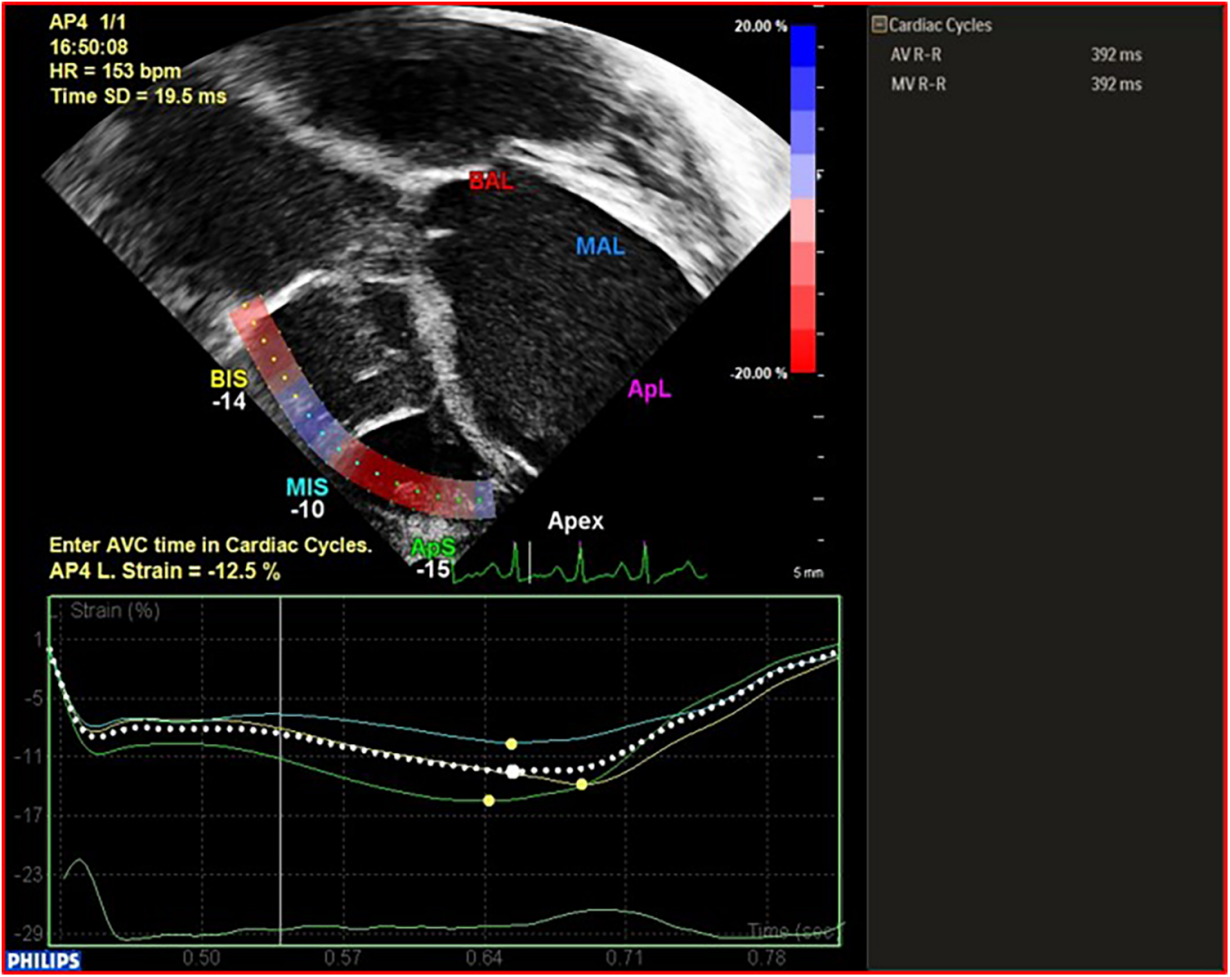
Example and calculation of RVFW-LS in a patient with BH EXCOR LVAD. RVFW-LS, right ventricular free wall longitudinal strain.

Specifically, in the pre-implantation phase, especially in small children with adequate acoustic windows, TTE offers a comprehensive evaluation of:

(1) Cardiac morphology
(2) Ventricular function: (a) **LV function** in children is assessed following the guidelines of the American Society of Echocardiography (ASE) (12), incorporating, when available, 3D evaluation; the measurement of the LV internal 2D dimension in the parasternal long-axis view at end-diastole (LVEDD) is crucial for LVAD candidates, enabling comparison and the assessment of unloading effects post-LVAD implantation. (b) **RV Function**: RV function represents a significant challenge during LVAD implantation, with failure rates reaching up to 60% in adults. Various mechanisms contribute to RV failure post-LVAD: firstly, the unloading of the LV and the subsequent leftward shift of the interventricular septum reducing the septal contribution to RV function; secondly, RV overload from the increased cardiac output induced by the LVAD. Moreover, many patients exhibit varying degrees of RV dysfunction before undergoing LVAD implantation. Evaluating RV function before starting ventricular assist support is crucial not only in adults but also in pediatric cases. This assessment is essential for appropriate patient selection and the identification of high-risk candidates who may require extended post-operative inotropic support or even necessitate RVAD implantation.
  Recent data from various international registries on mechanical circulatory support in the pediatric population have clearly demonstrated the correlation between RV failure and unfavorable outcomes in children (10, 13).
  Assessing RV function by echocardiography necessitates the incorporation of various parameters and technologies (12). Typically, RV systolic function in echocardiography is quantified by indices such as RV Fractional Area Change (RVFAC), tricuspid annular-plane systolic excursion (TAPSE), RV end-diastolic diameter/LV end-diastolic diameter ratio (RVEDD/LVEDD ratio), right atrial pressure (RAP) derived from inferior vena cava size and collapsibility. Additionally, recent advancements include strain imaging, measuring RV free-wall peak longitudinal strain (RVFW-LS) or RV global longitudinal strain (RV-GLS), reference values for the pediatric population are also reported for comparison (14, 15) Figures 2, 3. Ultimately, the estimation of systolic RV systolic pressure (PAPs) should be derived from the peak velocity of tricuspid regurgitation (TR) jet.
(3) Valve function, including aortic valve regurgitation (AR), which could reduce the effects of the LVAD in LV unloading or could be a contraindication to LVAD implantation; tricuspid regurgitation (TR) severity or the presence and mechanism of eventual mitral regurgitation (MR) or stenosis.
(4) Presence of intracardiac thrombi: does not contraindicate LVAD implantation; however, it may increase the likelihood of stroke during the procedure, necessitating carefully removal during surgery.
(5) Presence of any congenital heart disease: the presence of common anomalies like atrial septal defect (ASD) or patent foramen ovale (PFO), is relatively frequent in infants and necessitates correction during implantation. This is crucial because such anomalies can lead to significant hypoxemia through right-to-left shunting and paradoxical embolization during support. Similarly, any other intracardiac shunt, such as ventricular septal defects (VSD) or patent ductus arteriosus (PDA), must be identified by the echocardiographer and addressed at the time of implantation.

### 2.2 Peri-operative phase

In the perioperative setting, Transesophageal Echocardiography (TOE) offers real-time information in the cardiac operating room, both before the insertion of the device and after its placement. Furthermore, in instances of device malfunction or hemodynamic instability, TOE proves highly valuable in identifying the root cause of the problem.

TOE is therefore most commonly used to assess (2–9):

- RV function: it can be altered by the acute hemodynamic changes due to LVAD activation such as RV preload increment, RV afterload decrement and interventricular septum (IVS) leftward shifting.

Furthermore, the existence of post-implantation tricuspid regurgitation (TR) can contribute to RV dysfunction and low cardiac output state. Hemodynamic data should encompass assessments of RV systolic pressure through TR peak velocity by Doppler.

- Intracardiac shunts: in particular, the presence of a PFO- if not identified by TTE at pre-implant evaluation- should be assessed before surgery (usually in the operating room, this involves the administration of intravenous agitated saline in conjunction with a properly executed Valsalva maneuver) and after cardiopulmonary bypass with the evaluation of the shunt direction that can be inverted after LVAD starting, leading to paradoxical embolization or hypoxemia.
- De-airing before the LVAD is activated: as air bubbles are lighter than blood, they tend to move toward the anterior chest wall and along the anterior aortic root. This migration may lead to embolization in the right coronary artery, potentially causing ischemia and significant RV dysfunction.
- Adequate positioning of cannulas: aligning the LVAD inflow cannula within the LV apex with the mitral valve orifice is crucial. This alignment helps ensure appropriate unloading, prevents suction-related issues, and mitigates the risk of damage to the mitral valve.
- Appropriate IVS position to adjust for the pump speed assuring an adequate LV unloading without RV enlargement: usually during implantation phase, it is recommended to ensure the septum is in a neutral position. This helps achieve a balanced unloading of the LV without inducing excessive dilation of the RV. The leftward shift of the IVS is usually more pronounced in case of hypovolemia, RV dysfunction or substantial TR prior to LVAD implantation. Patients with increase LVAD flow often exhibit a leftward IVS leading to the so called “suction events”, which usually resolves after decreasing LVAD pump flow. These events can be easily recognized by echo (Supplementary Material Avi File S1, S2, S3, S4).
- LV sizes, Mitral Regurgitation (MR), Aortic Regurgitation (AR), aortic valve opening.

The implantation of an LVAD results in a decrease in LV size, enhancement of mitral valve leaflet coaptation, and typically a reduction in preexisting mitral regurgitation (MR). Persistence of MR after LVAD insertion may suggest insufficient LV decompression. The absence of decompression following LVAD implantation, coupled with a rightward shift of the septum, should prompt consideration of potential issues such as suboptimal LVAD support, abnormal device function, or cannula obstruction. The absence of decompression following LVAD implantation, coupled with a rightward shift of the IVS, should prompt consideration of potential issues such as suboptimal LVAD support, abnormal device function, or cannula obstruction.

- Aortic valve regurgitation could appear or could be worsened by the presence of LVAD leading to a decrement of the total cardiac output because of recycling. Aortic valve opening is fundamental to prevent ventricular thrombosis and to promote native heart contraction.

### 2.3 Post-operative phase

Combined information (TTE + TOE) are often used in the post-operative settings to study at pre-specified intervals (usually once a week/every 2 weeks in children with BH LVAD EXCOR) during long-term support: (1) native heart functioning during support, (2) VAD components functioning, (3) VAD parameter optimization, (4) VAD related complication or problem-focused echocardiography, (5) heart recovery (4).

(1) **Analysis of the native heart functioning** during support need a routinely assessment of:
  - AR which can cause wasteful recirculation reducing systemic perfusion. The likelihood to developing AR is elevated during prolonged LVAD support due to the increased pressure gradient across the aortic valve. This higher transvalvular aortic gradient develops because of LV decompression and consequent fall in left ventricular end-diastolic pressure.
  - MR is often signiﬁcantly reduced after LVAD support, however the persistence of severe MR following LVAD placement may suggest insufficient LV decompression. Additionally, MR can gradually intensify in the later stages post-LVAD implantation due to the reduced LV unloading, possibly resulting from factors such as the need for LVAD reprogramming.
  - Systolic Pulmonary arterial pressure assessment from doppler peak velocity TR, as it may normalize following LVAD support, thus enabling eligibility for transplantation at a later date.
  - LV unloading, RV function and the interventricular septum position: following the placement of an LVAD, there is typically a reduction in LV end-diastolic diameter (LVEDD), and there is potential improvement in the Ejection Fraction (EF). Changes in RV function occur due to increased preload and decreased afterload. In the case of proper LVAD settings, it is advisable for the interventricular septum (IVS) to be in a neutral position.

Ejection Fraction and LVEDD: the evaluation of EF is challenging during LVAD support, especially in pulsatile-flow VADs it is difficult to extrapolate the intrinsic ejection and contractility of LV because the unloading by the VAD and the asynchronous modality of the BH and the native heart. Usually a calculation of EF by biplane Simpson Method and LVEDD among at least 5 cardiac cycles is necessary to obtain a reliable estimation of EF in these patients, given the rare opening of the aortic valve during stable support. LVEDD is usually indexed to body surface area in children and need to be expressed in Z-score (12). After an initial phase of LVEDD reduction due to LV unloading, the LVEDD may gradually increase in the later stages following LVAD implantation due to insufficient unloading and the requirement for LVAD reprogramming or the need for larger BH chamber size because of patient growth (34).

**Figure 4:**
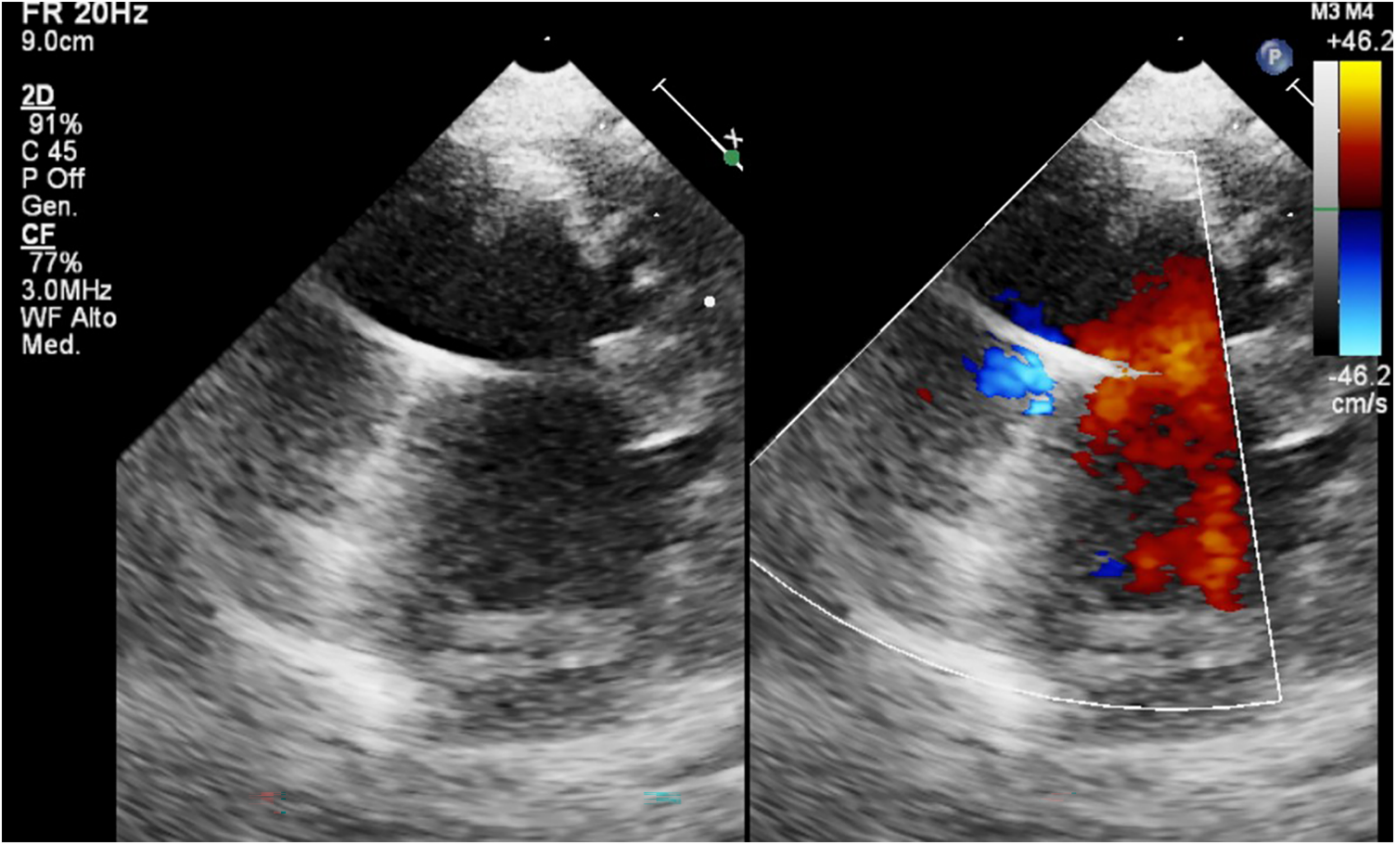
BH inflow cannula shown in parasternal short axis view, with color Doppler interrogation of cannula flow.

**Figure 5:**
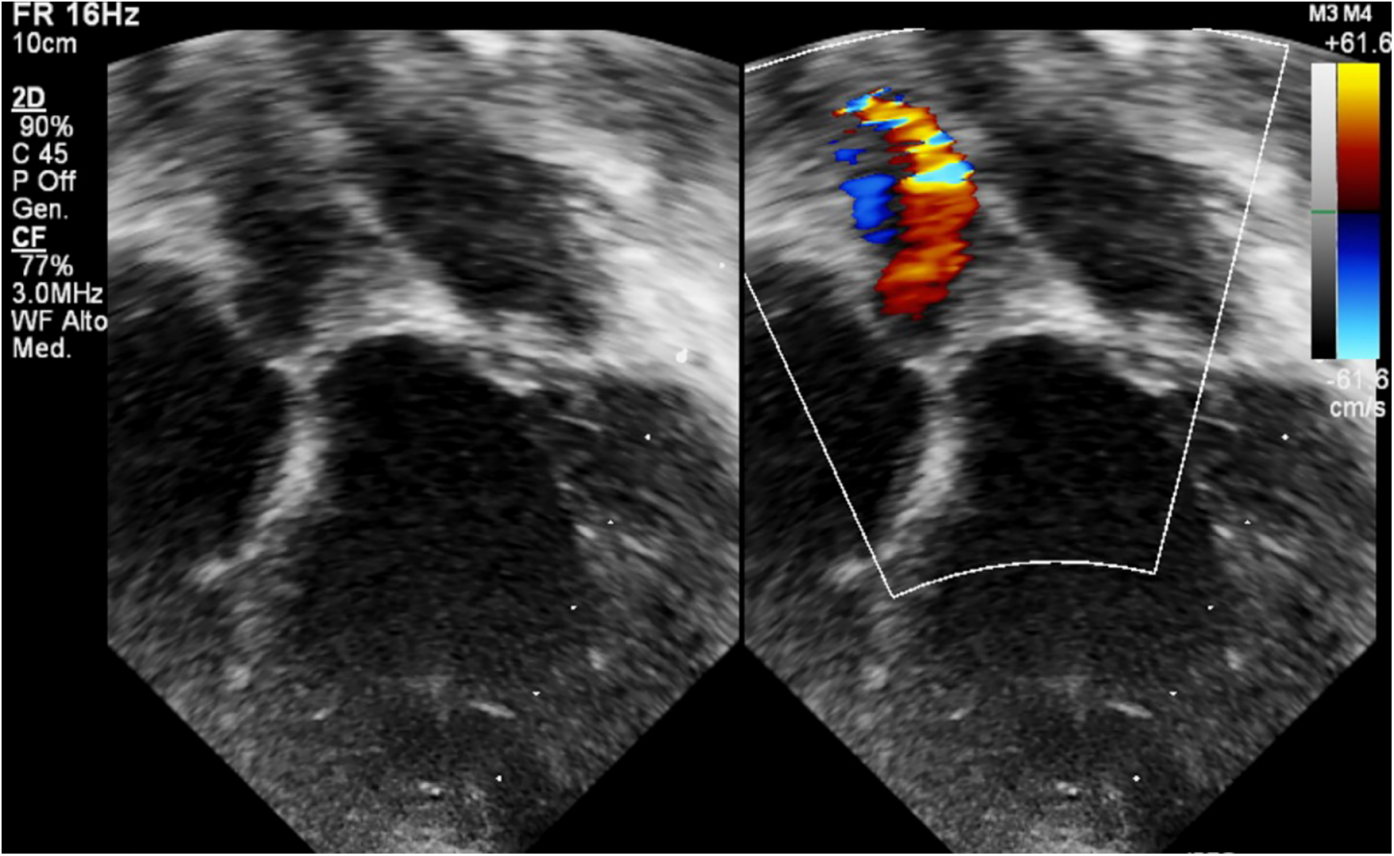
BH outflow cannula showing color Doppler flow in ascending aorta.

**Figure 6:**
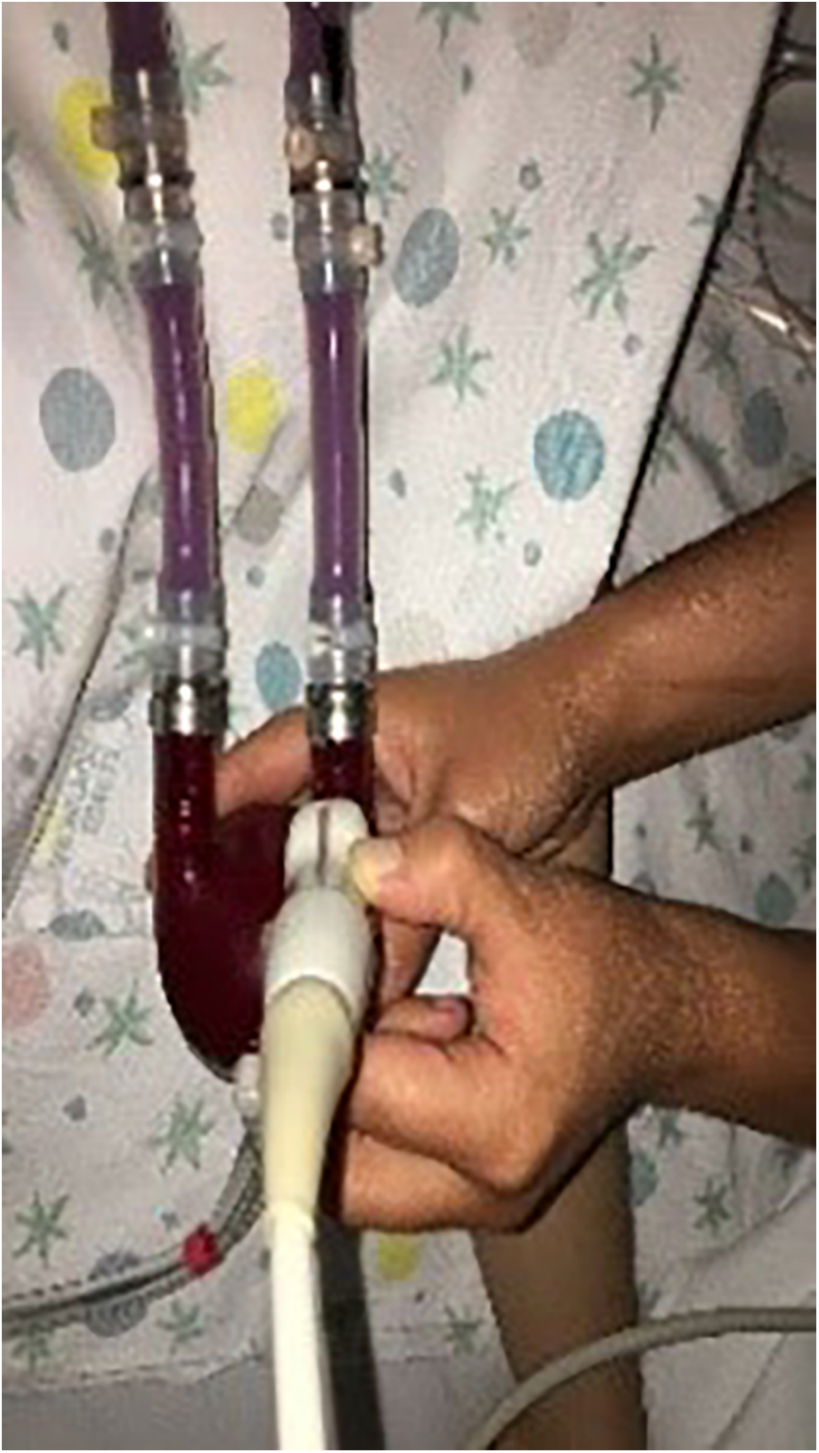
Echocardiographic evaluation by direct positioning of the transducer on BH components (inflow & outflow valves and membrane).

**Figure 7:**
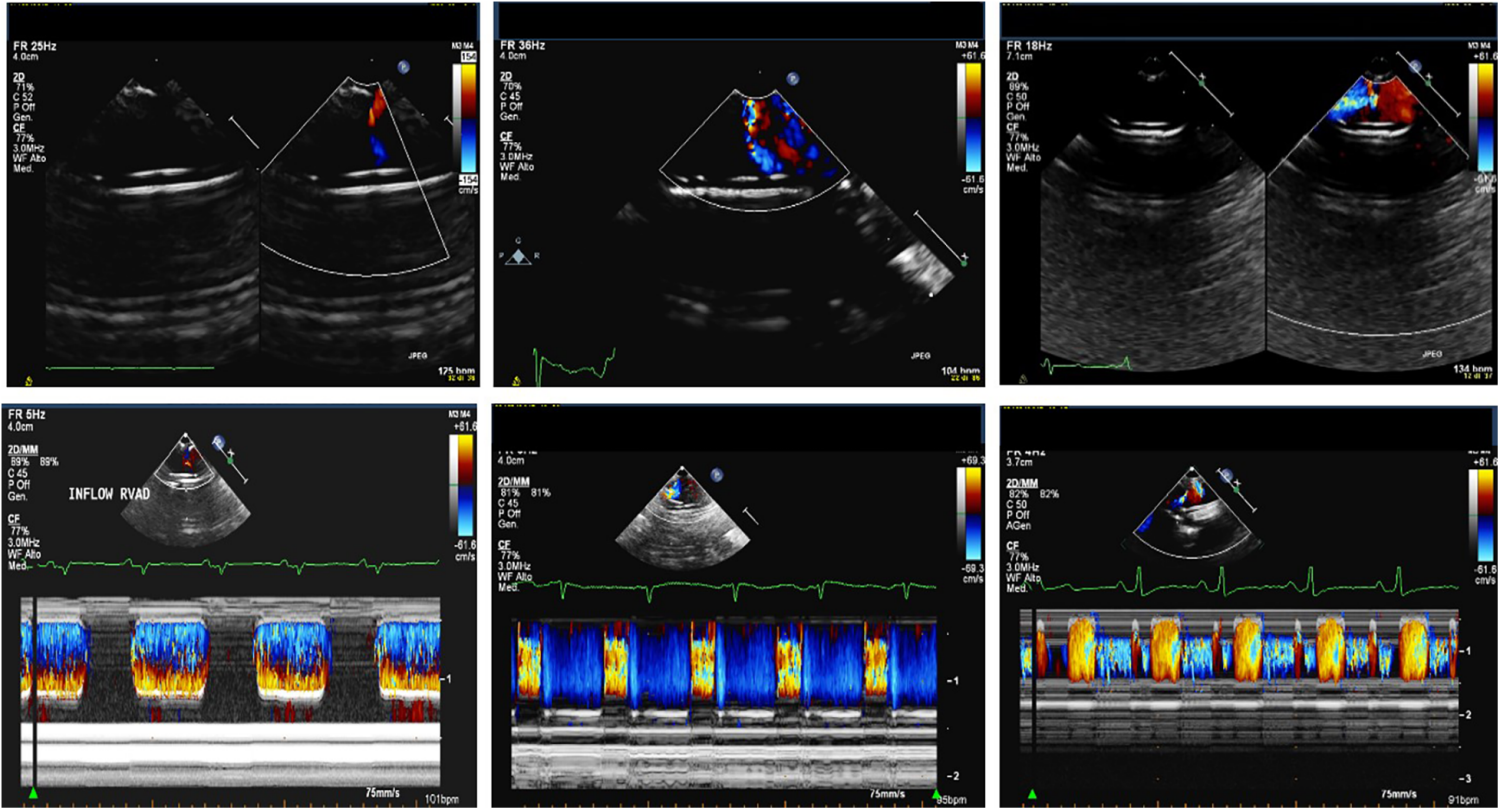
2D color and M-mode color Doppler of no regurgitant Berlin heart valve (left), a Berlin heart valve with a mild regurgitation (middle) and a Berlin heart valve with a moderate regurgitation (right).

**Figure 8:**
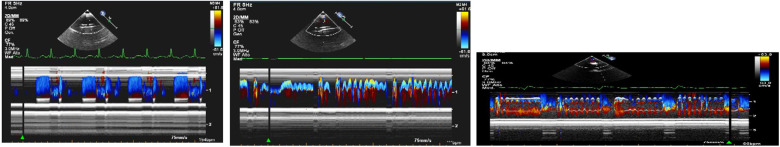
M-mode color Doppler images of Berlin heart valves illustrate a common beat phenomenon pattern (left panel), the beat phenomenon observed during in atrial fibrillation (middle panel) and the beat phenomenon occurring in a case of cannula compression (right panel).

(2) **VAD components functioning**: After LVAD placement, mechanical dysfunction can be occasionally observed. Dysfunctions of the device can arise from either the failure of the VAD system components themselves or from pathophysiological changes that interfere with the proper functioning of the VAD. Echocardiography could be useful to study VAD components and to identify VAD malfunctioning (2–4).
  - VAD Cannulas: the visualization of the inflow cannula and its orientation can be achieved through 2D echo or 3D echo imaging, providing a clearer depiction of the relationship between the inflow cannula, IVS, and mitral valve (See Figure 4). Typically, the inflow cannula originates from the LV apex and, in such cases, it should align with the LV inflow tract, coinciding with mitral valve opening, and avoiding abnormal contact with any wall. Abnormally high velocity and turbulent flow may indicate obstruction of the inflow cannula such as the presence of thrombus, suction issues, or reverse remodeling causing an LV cavity reduction.
  - Doppler allows the detection of gradient on the inflow/outflow cannulas: a suggested indication of normal flow into the LV cannula during filling of pulsatile flow LVADs is a peak velocity below 2.3 m/s in adults (19). These cutoffs are not reliable in small children due to the smaller cannula size and faster heart rate, so there are no established standard doppler flow velocity benchmarks for the inflow and outflow cannulas in small children supported by pulsatile-flow VADs. Any difference in doppler peak velocity over time in the same patients could help in detecting new onset of cannula obstruction through serial echocardiographic evaluation during surveillance examination. The outflow cannula is typically located in the right anterolateral aspect of the ascending aorta (Figure 5). With sufficient acoustic windows, a long axis view of the ascending aorta usually reveals the anastomosis of the outflow cannula to the ascending aorta. Regurgitation at the outflow valve is characterized by retrograde flow observed within the outflow graft during LVAD diastole potentially resulting in decreased systemic perfusion.
  - Inﬂow Valve Regurgitation: is the predominant reason for LVAD dysfunction. In cases of inflow valve regurgitation, echocardiography may reveal an inadequately decompressed LV, frequent opening of the aortic valve and a decrease of the velocity time integral and peak velocities of the outflow graft. The pulsed doppler pattern of the inflow cannula typically displays notable fluctuations in the flow of inflow valve regurgitation concerning the native cardiac cycle.
  - VAD Chamber: Due to the inherent echogenicity of the device, visualizing thrombi within the pump using echocardiography is not feasible. Thromboembolic material may form and become detectable in the left atrial appendage or in the LV apex impeding forward flow. It is also crucial to rule out any obstruction in the inflow and outflow cannula, as well as other sources of thromboembolism. In pulsatile VADs, membrane alterations could contribute to this phenomenon (20).
  - BH membrane and valve Echo Evaluation: the functioning of BH device and its interaction with the native heart can be assessed using echocardiography in the pediatric population. In our publication (33), we shared our insights into a novel method for echocardiographic assessment of BH membrane movement, inflow and outflow valves. This evaluation was conducted using 2D, 2D-color Doppler, M-mode, and M-mode color Doppler. Our approach involved placing the transducer directly on the BH inflow and outflow cannulas to observe the movement of valve leaflets in a long axis view (Figure 6) with the aim of directly studying the device's functionality. Simultaneously, the transducer was positioned at the center of the VAD chamber to evaluate its movement. In our limited experience, we noted that mild regurgitation of BH valves is frequently observed and can be tolerated (see Figure 7). Conversely, whenever we identified moderate to severe regurgitation, we could attribute it to specific events such as Berlin Heart malfunction, patient tamponade, cannula compression, or patient arrhythmias. (Figure 8). Therefore, a systematic echocardiographic assessment of Berlin Heart components can aid in diagnosing specific cases, contribute to BH programming, and facilitate the identification of both normal and anomalous interactions between the VAD and the native heart.
(3) **VAD optimization**: echocardiographic measurements is needed during adjustments of VAD parameters in pulsatile-flow VADs (VAD rate, systole duration, filling and ejection pressure) to guide BH device settings. In instances of inadequate ventricular unloading, the flow of the LVAD can be increased until the chamber size decreases or the septum ceases to bow into the RV. If the ventricle is underfilled and there is obstruction of the inflow cannula, the LVAD flow can be reduced until septal neutrality is achieved or the chamber enlarges to the point that the cannula is no longer obstructed, as observed in 2D imaging or when Doppler velocities return to normal levels. Adjustments to the LVAD parameters can be made to allow a slight opening of the aortic valve preventing thrombus formation in the aortic root. In addition, VAD chamber size should be increased to assure an adequate LV unloading according to patient growth (34).
(4) **VAD related complication or problem focused-echocardiography**

**Figure 9:**
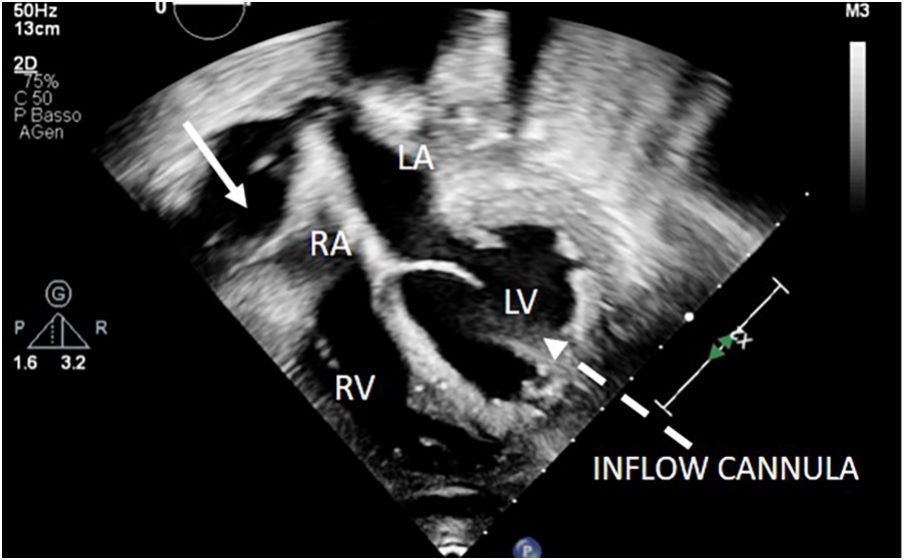
2D apical 4C view showing right atrium collapse due to retroatrial effusion after LVAD implantation. LVAD inflow cannula: dotted arrow. Retroatrial effusion: arrow.

**Figure 10:**
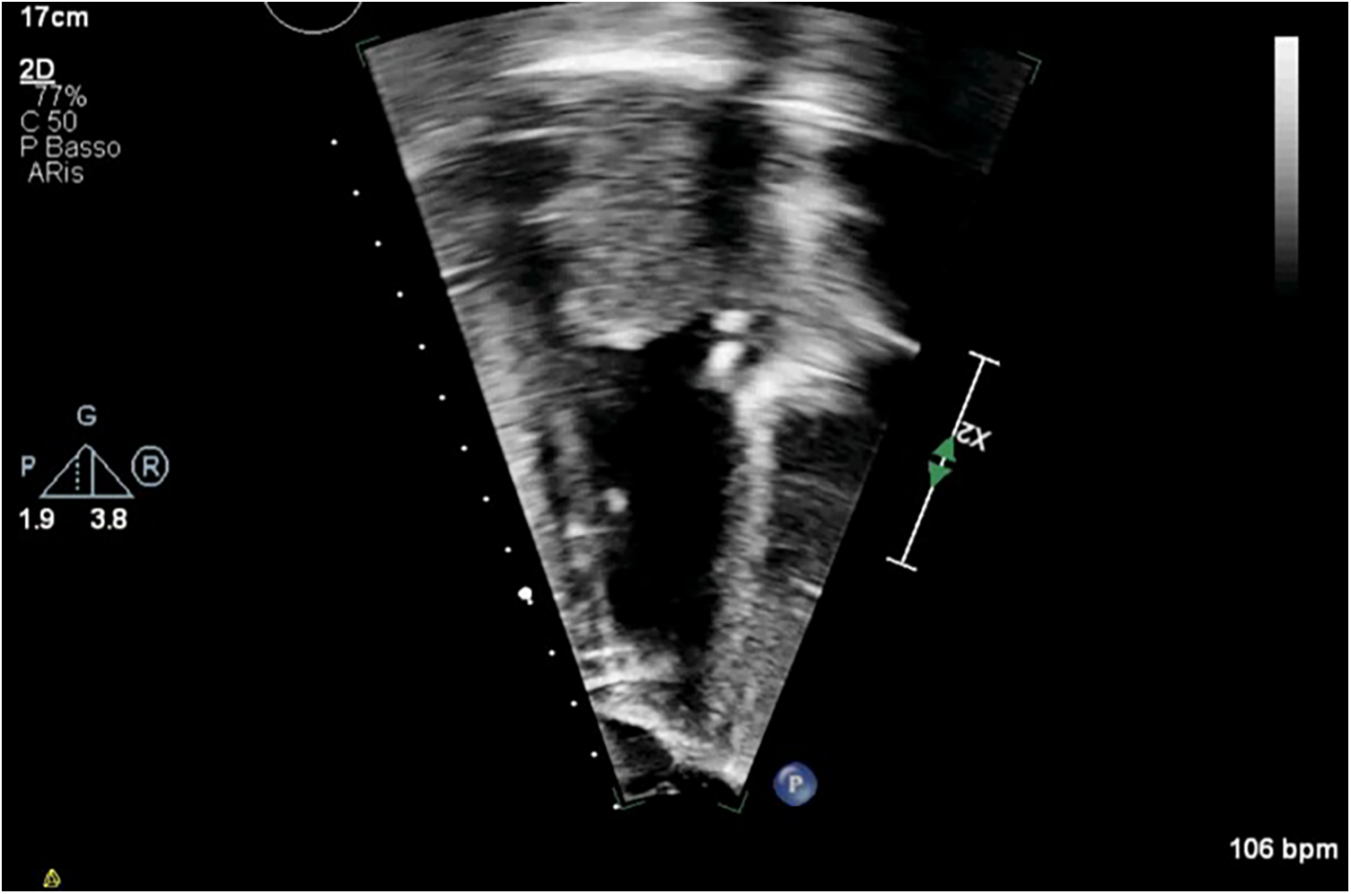
2D apical 4C view showing a huge right retroatrial hematoma after LVAD implantation.

Following surgery, the occurrence of hemodynamic instability necessitates comprehensive consideration of potential factors, such as:

- Hypovolemia should be entertained when both the RV and LV cavities appear diminished. Acute RV dysfunction may present as a dilated and hypokinetic RV accompanied by substantial functional TR, a small LV, and intermittent obstruction of the inﬂow cannula due to the collapsed LV.
- Cardiac tamponade: stands out as a prevalent cause of hemodynamic instability following the insertion of LVAD. Echocardiographic indicators consistent with tamponade include systolic collapse of the right atrium and diastolic collapse of the RV. This condition may arise from bleeding, pericardial effusion, or hemopericardium (Figures 9, 10)
- Aortic dissection (very rare in small children): echocardiography provides the capability to visualize intimal flaps or distinguish between true and false lumens. Aortic dissection may be induced by an LVAD due to elevated shear stress on the aortic intima resulting from the high velocity blood ejection against the aortic wall.
- Thrombosis of LVAD should be suspected when there is a combination of signs, such as the rightward deviation of the IVS, functional MR accompanied by annular dilation, aortic valve opening in every cardiac cycle, spontaneous contrast in the left atrium and/or LV, and regurgitant ﬂow through both cannulas.
- VAD endocarditis poses a significant risk of morbidity and mortality following LVAD implantation, frequently linked to mechanical complications of the the device. Echocardiographic indicators indicative of VAD endocarditis encompass the identification of echodense structures resembling vegetations on the inflow or outflow cannulas, as well as on native or prosthetic valves; Additionally, signs may include LVAD inlet obstruction, malfunction of the inflow and outflow valves, and rupture of the outflow cannula (35).

When the HF team suspects an issue with LVAD function, it is recommended to perform a problem-focused echocardiography exam. It is typically guided from the following indications: an alarm from the driving unit, emergence of new symptoms, suspicion of hemolysis or infection, onset of new arrhythmias, hypotension, or other clinical tests suggesting LVAD malfunction. In order to enhance the effectiveness and relevance of a troubleshooting echocardiographic exam, the HF team should communicate the specific indications for the study to the echocardiographer.

It is crucial to emphasize that in certain instances, echocardiography may not yield definitive results in identifying complications such as inflow-cannula malposition, pump thrombosis, outflow-graft kinking or obstruction, intra- or extracardiac thrombus, or cardiac compression due to a pericardial or extra-pericardial hematoma. In such scenarios, cardiac computed tomography (CCT) can offer valuables supplementary information for a more conclusive diagnosis (21).

(5) **Heart recovery:** during LVAD support, reverse cardiac remodeling and subsequent heart recovery can occur. In contrast to continuous-flow VADs utilized in adults, pulsatile-flow VADs enable a thorough evaluation of heart function even when the pump is completely stopped (22, 23).

Echocardiography plays a pivotal role in evaluation cardiac recovery, facilitating the identification of potential candidates for weaning, aiding in the decision-making process regarding the removal of VAD. This evaluation includes patients exhibiting signs of significant reverse remodeling and apparent improvement in contractile function.

Echocardiographic parameters suggesting myocardial recovery are: EF >=45%, LVEDD <45 mm or with a *Z*-score <+ 2 in the pediatric population, RVFAC higher than 40%, none or mild heart valve dysfunction. When considering recovery, usually the echocardiographic study includes the measurements of LV size and function. These assessments are conducted with gradually reducing the level of support; the final step consists in stopping the BH pump during an off-pump trial in the catheterization lab (23) (Supplementary Material Avi File S5). Measurements of LVEDD can either decrease indicating recovery or increase, evidencing a failed wean. Simultaneously, there might be an increase or decrease of EF or valve regurgitation during the pump-off trial, reflecting the ventricle's capacity to respond to augmented preload.

### 2.4 Ventricular Mechanics during LVAD

Several emerging echocardiographic techniques may have a clinical impact on the monitoring of patients supported by LVADs. For instance, strain measurements conducted through 2D-Speckle tracking offer a more detailed understanding into the intrinsic cardiac functions of both ventricles when compared to traditional echocardiography. Specifically, RV LS can be employed for a non-invasive assessment of RV systolic function prior to and after LVAD implantation (24, 25).

Strain imaging offers several advantages, including its capacity for providing an objective and quantitative assessment of both global and regional movement in myocardial segments. It is angle-independent and enables the quantification of intra-ventricular asynchrony and dyssynergy. Moreover, 2D strain analysis is relatively independent of RV preload and afterload.

RV-GLS and RVFW-LS have showed important clinical implications and an increasing role for adult patients necessitating long-term LVAD support (16, 26–28).

As reported by our group (18, 28), also in children with good acoustic windows, standard acquisition of 2D speckle tracking indices of LV GLS, RVFW-LS should be routinely assessed before and during LVAD support. While the positive impacts of LV unloading provided by LVAD are well-established, the effects of LVAD on RV function are still a matter of debate. The RV shares muscle fibers, the IVS and the pericardial sac with the LV, resulting a complex interplay between the two ventricles. Although there is a lack of literature data comparing the long-term effects of pulsatile-flow vs. continuous-flow LVAD in pediatric patients, there is the speculation that the time limited effect on LV and RV unloading may be associated with pulsatile-flow LVAD. In adult patients treated with continuous-flow LVAD, a progressive improvement in RV function has been observed up to the 6-month follow-up (25, 29).

The assessment of ventricular unloading and mechanics during LVAD support is particularly intriguing due to the demonstrated correlation between ventricular unloading, heart remodeling and myocardial recovery (30, 31).

## 3 Discussion

Echocardiography is a fundamental tool in the evaluation patients undergoing VAD implantation (2–8). Currently, there is a scarcity of reports are very limited on the utilization of echocardiography for assessing pediatric pulsatile-flow VADs, due to the low case volumes at every cardiac pediatric center. Because of its unique nature, pediatric population and pulsatile-flow LVADs used in pediatrics require specific skills so that pediatric echocardiographers must develop a systematic approach in order to image the patients pre and post LVAD implantation and have prompt knowledge of serial echocardiographic evaluation and eventual complications during LVAD support. The growing use of VADs in the pediatric population is accompanied by an increased reliance on TTE and TOE both before, during and after implantation. While it can be assumed that the main principles of echocardiographic imaging for LVADs apply to both pediatric and adult patients, the diverse array of VAD models, each with distinct principles, necessitates a tailored echocardiographic assessment that aligns with the characteristics of the specific implanted device.

Few data are available regarding the LVAD pediatric population. The larger study was proposed by Sachdeva et al, who reported data of 32 patients (median age, 3 years and median weight, 12.3 kg) treated with LVAD (20) and BiVAD (12) for a median time of 12 days. They underlined the importance of echocardiography, particularly to assess RV function, intracardiac shunt (that was found in 11 patients), intracardiac thrombus (1 patient), cardiac dimension, valves insufficiency, post-implantation pericardial effusion (found in 16 patients) or hematomas (found in 12 patients) (11).

Several researchers discussed the application of strain and strain rate in the LVAD population. However, the majority of these studies primarily concentrate on investigating predictors of RV failure following LVAD implantation. Importantly, all of these studies are focused around adult patients with CF VADs (16, 17, 24–27).

Experiences on longer LVAD staying in pediatric population have been recently reported (18, 28, 32) showing that during PF-LVAD support, LV dimensions are reduced due to reverse remodeling with an improvement of LV function at short term follow up, with a decreasing effect of the level of unloading over time. The findings indicates that the advantages conferred by the LVAD diminish over time for both LV and RV. Specifically, an increase in LVEF corresponds to an improvement in RVFAC at 1 and 3 months of follow-up, while a decline of LVEF is associated with a decrease in RVFAC at 6 months. Similarly, an increase in LV-GLS precedes an increase of RVFW-LS at 1 month, while a deterioration in LV-GLS precedes a decline in RVFW-LS at 6 months. These results underscore the role of biventricular interaction during LVAD support and emphasizes the necessity for ongoing LVAD reprogramming to ensure an appropriate support.

## 4 Conclusion

In conclusion, echocardiography serves as the primary imaging modality for the selection and monitoring of pediatric patients with LVADs. It enables to study the VAD-heart interaction in the pre-implantation, peri-implantation, post-implantation phase and to assess the potential of myocardial recovery. Dedicated physicians and protocol should be present in VAD programs to maximize the benefits of the VAD therapy. Several issues remain unsolved and need further study such as: the cause of the reduction in unloading and the decline in RV function during the long term follow up, the role of LVAD continuous reprogramming, the improvement of BH valve functioning, the standardization of a weaning protocol to identify pediatric patients who can show a stable cardiac recovery post LVAD removal.

## Author contributions

RI: Conceptualization, Writing – original draft. AD: Writing – review & editing. AA: Supervision, Writing – review & editing.

## Supplementary material

Supplementary Material AVI File S1 TOE 4C view: example of suction event in the perioperative implantation phase, note the leftward shift of the IVS.

Supplementary Material AVI File S2 TOE 4C view: resolution of suction event in the same patient after correcting hypovolemia.

Supplementary Material AVI File S3 2D apical 4C view showing a virtual LV cavity due to suction event.

Supplementary Material AVI File S4 TOE apical 4C view evidencing LV suction with a virtual LV cavity mimicking LA thrombosis, note the leftward IVS shift.

Supplementary Material AVI File S5 2D apical 4C view assessment of RV and LV function during pump off trial at 60 min after pump stop.
